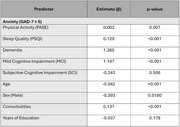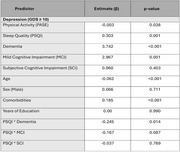# The Impact of Physical Activity and Sleep Quality on Risk of Anxiety and Depression across Cognitive Statuses in Older Adults

**DOI:** 10.1002/alz70858_103509

**Published:** 2025-12-24

**Authors:** Maral Ghodsi, Laura E. Middleton, Carrie A McAiney, Heather Keller

**Affiliations:** ^1^ University of Waterloo, Waterloo, ON, Canada

## Abstract

**Background:**

Anxiety and depression are common among individuals with mild cognitive impairment (MCI) and dementia. These conditions are associated with accelerated cognitive decline, increased caregiver burden, and reduced quality of life. Given the adverse effects of pharmacological treatments, non‐pharmacological approaches are gaining attention. Physical activity and sleep quality have shown promise in reducing the risk of anxiety and depression, but their potential benefits remain largely underexplored in people with cognitive impairment. The objective here is to explore the effects of these modifiable risk factors on the likelihood of screening positive for anxiety and depression across different levels of cognitive status in older adults.

**Method:**

This is a secondary analysis of the Comprehensive Assessment of Neurodegeneration and Dementia (COMPASS‐ND) study. Physical activity and sleep quality were assessed using the Physical Activity Scale for the Elderly (PASE) and the Pittsburgh Sleep Quality Index (PSQI), respectively. Anxiety and depression symptoms were assessed using the Generalized Anxiety Disorder 7–item (GAD‐7) and the Geriatric Depression Scale (GDS), with cutoff scores of GAD‐7 ≥ 5 and GDS ≥ 10 indicating at least mild anxiety and depression, respectively. Logistic regression models with backward selection of covariates were used to explore the presence of these conditions.

**Result:**

The study included 956 participants with no cognitive impairment (CU, *n* = 150), subjective cognitive impairment (SCI, *n* = 116), mild cognitive impairment (MCI, *n* = 460), or dementia (*n* = 230). In regression analyses, physical activity (PASE score) was associated with lower odds of anxiety (*p* = 0.007) and depression (*p* = 0.038); however, this association did not vary across cognitive status levels. In contrast, sleep disturbances (PSQI score) were strongly associated with increased odds of both anxiety (*p* < 0.001) and depression (*p* = 0.001), with the effect on depression being particularly pronounced among individuals with dementia (PSQI * dementia interaction, *p* = 0.014).

**Conclusion:**

This study suggests that both sleep quality and physical activity may be protective factors against anxiety and depressive symptoms among older adults with varying levels of cognitive impairment. Future research should examine the effects of physical activity and/or sleep interventions on anxiety and depression among people with mild cognitive impairment and dementia.